# Association between stress types and adolescent suicides: findings from the Korea Youth Risk Behavior Survey

**DOI:** 10.3389/fpsyt.2024.1321925

**Published:** 2024-07-08

**Authors:** Soo Young Kim, Yu Shin Park, Hye Jin Joo, Eun-Cheol Park

**Affiliations:** ^1^ Department of Public Health, Graduate School, Yonsei University, Seoul, Republic of Korea; ^2^ Institute of Health Services Research, Yonsei University, Seoul, Republic of Korea; ^3^ Department of Preventive Medicine, Gachon University College of Medicine, Incheon, Republic of Korea; ^4^ Department of Preventive Medicine, Yonsei University, College of Medicine, Seoul, Republic of Korea

**Keywords:** teenage suicides, cause for adolescent suicides, types of stress among adolescents, suicidal ideation, suicide attempts

## Abstract

**Objective:**

This study aims to explore the association between types of stress and suicidal behaviors—ideation and attempts—among Korean adolescents in two distinct years: 2015 and 2020.

**Methods:**

Data were acquired from the Korea Youth Risk Behavior Web-based Survey conducted in 2015 and 2020. Participants’ desire for suicide was evaluated by asking questions about suicidal ideation and attempts, during the past year. Types of stress were divided into five categories: none, home, school, academic achievement, and appearance. Multiple logistic regression was used to investigate the association between variables of interest and dependent variables.

**Results:**

Among the 77,363 adolescents included in this study, 8.8% male and 13.2% female participants had seriously thought about committing suicide, and the rate of male and female participants who tried committing suicide was 1.6% and 2.5%, respectively. While every type of stress was highly associated with suicidal ideation, family and home types of stress had the highest odds ratio (OR), that was statistically significant for both sexes (Male: OR 3.81, 95% Confidence Interval [CI] 2.81 to 5.15; Female: OR 3.64, 95% CI 2.42 to 5.50). Moreover, the OR increased in order of: appearance; academic achievement; school and friends; and family and home, compared to the group that perceived no stress. Suicidal attempts were statistically significant and higher in likelihood, only amongst the female group that experienced stress from family and home (OR 2.48, 95% CI 1.08 to 5.67). In comparison to year 2015, suicidal ideation and attempts decreased in year 2020, but participants experiencing stress from family and home had a higher tendency of attempting suicide, though not statistically significant, regardless of their sex (Male: OR 1.03, 95% CI 0.74 to 1.44; Female: OR 1.06, 95% CI 0.81 to 1.4).

**Conclusion:**

Adolescents who experience stress from family and home, or school and friends, are more likely to think about suicide, or carry it out, as compared to those having different causes of stress.

## 1 Introduction

Adolescence is a stage of life marked by crucial changes—physical, emotional, and behavioral development—that are highly influenced by the surrounding environment, at a particular point in time (1). Recent studies found that adolescence has been prolonged, due to early puberty and delayed timing of adulthood (2). Adolescence is a period when both physical and mental state transits, and there are many common and provisional mental illness that develop at this time such as depersonalization, body dysmorphic disorder, or anorexia. Owing to such chaotic adjustments that teenagers have to go through during adolescence, mental problems that may lead to suicide, have become a social issue in many countries (3, 4). A national representative survey of 10,123 adolescents aged 13 to 18 years conducted in the United States, reported a 31.9%, 19.1%, and 14.3% prevalence for anxiety, behavior, and mood disorders, respectively (5). The World Health Organization estimates that 62,000 adolescents died in 2016 as a result of self-harm, which is the third leading cause of death among those aged 15 to 19 years (6). Moreover, in the United States, the suicide rate among youth aged 10 to 24 years increased 56% between 2007 and 2017, according to reports from the Centers for Disease Control and Prevention (7).

From Human Birth Theory by Massimo Fagioli, birth itself is equally healthy state for all individuals, and mental disorders develop exclusively due to postnatal experiences, especially relationship with others from birth to 1 year (8). According to this theory, individuals carry both vitality and annulment at birth. However, depending on the sufficiency of affection post-birth, vitality can be diminished and annulment drive is initiated. This drive is a non-conscious absence of affectivity, and this represents the main factor of mental illness developed later (9). Adolescence is a period of life when we realize and start to build identity of our own. Those who have let down the vitality from lack of caregivers’ affection and safe environment at newborn period may have easily triggered development of mental disorders in adolescence.

In addition to vulnerabilities caused by effects of physical and mental changes, most adolescents are challenged by adverse environmental factors—conflicts with parents, familial discord, academic stress, and school bullying. Previous studies have investigated the factors related to adolescents’ mental health with regard to specific stressors, such as peer rejection and victimization (10), conflicts with parents (11), parental discord (12), and academic failure (13). Stress coming from these sources, particularly from the surrounding environment, have a greater influence on youth than adults (14). Therefore, it is crucial for suicide prevention policies to include measures specifically targeted in coping with such stress factors that teenagers encounter.

Korea ranked first in suicide rates among the countries considered, with a 24.6% mortality rate per 100,000 persons, as per the 2019 update (15). Based on an Organization for Economic Cooperation and Development (OECD) report, suicide rates decreased in many countries, including Canada, Finland, and Switzerland, whereas suicide rates greatly increased in Korea during the 1990s to 2017 (16). According to the Korea National Statistical Office, the youth suicide rate increased from 7.7 per 100,000 persons in 2017 to 9.1 per 100,000 persons in 2018 (17). Among OECD member nations, Korea has the highest suicide rates; hence, there have been various studies on suicide, focusing on the different age groups that are prone to suicide (18–20).

Stress is a high potential trigger for adolescents to commit suicide. Given that Korea contributes to a large chunk of the world’s suicide rates, this study will divide stress into categories to identify which stress factor is the most influential. During the pandemic era, many students went through surrounding environmental changes, such as taking online classes and spending a majority of time at home. Thus, when using year 2020 data for analyzing stress of adolescents, comparative studies should be conducted, using previous years’ data. This study’s aim is to explore the association between types of stress and suicidal behaviors—ideation and attempts—in Korean adolescents during 2015 and 2020.

## 2 Method

### 2.1 Data

Data used in the study were obtained from the 2015 and 2020 editions of the Korea Youth Risk Behavior Web-based Survey (KYRBS)—a nationwide survey conducted annually to evaluate Korean adolescents’ health behavior status—established in 2005 by the Korea Center for Disease Control and Prevention Agency. Participants of year 2015 and 2020 were included in the sample because the question about most affected stress type was asked in those two years. The surveys’ data are used to monitor and estimate the prevalence of diseases in relation to several health factors in the South Korean youth population (21–23).

### 2.2 Participants

A total of 122,991 adolescents, currently enrolled in Korea’s middle and high schools, responded to the survey. The sample includes adolescents aged 13 to 18, which correspond to middle and high school students in Korean curriculum. In 2015, 797 schools and 68,043 students participated in the survey, and 793 schools with 54,948 students participating in 2020. All schools involved in the survey are located in the 17 regional districts of Korea including metropolitan and rural areas. There were no variable available in the dataset where the schools are located, but students’ residential area was asked, and so we used that variable to set different regions per student. Students were not forced to answer the web-based questionnaire, but they were informed and given time to fill out during class period. Engagement rate was high, average of about 96%, and therefore including 122,991 participants before eliminating those who are not qualified for the study. After adjusting the variables of interest and dependent variables, 116,520 adolescents were included in the study. Those who chose “other than above,” as the answer to the question on type of perceived stress and instead wrote freely and descriptively about their stress, were excluded. After eliminating the missing variables and adding covariates, including confounding variables that could affect the main variables, 77,363 participants were finally included.

Institutional Review Board approval or participants’ informed consent were not required because this study used data from the KYRBS, a secondary dataset accessible to the public, that does not include private material.

### 2.3 Variables

The two dependent variables related to the concept of suicidal behavior were: suicidal ideation and attempts. The question asked was whether the participants had seriously considered committing suicide, and actually tried to kill themselves in the past year, for which, the response choices were either “yes” or “no.” As suicidal ideation and attempts differed in the severity of desire to commit suicide, two dependent variables were considered.

**Figure 1:**
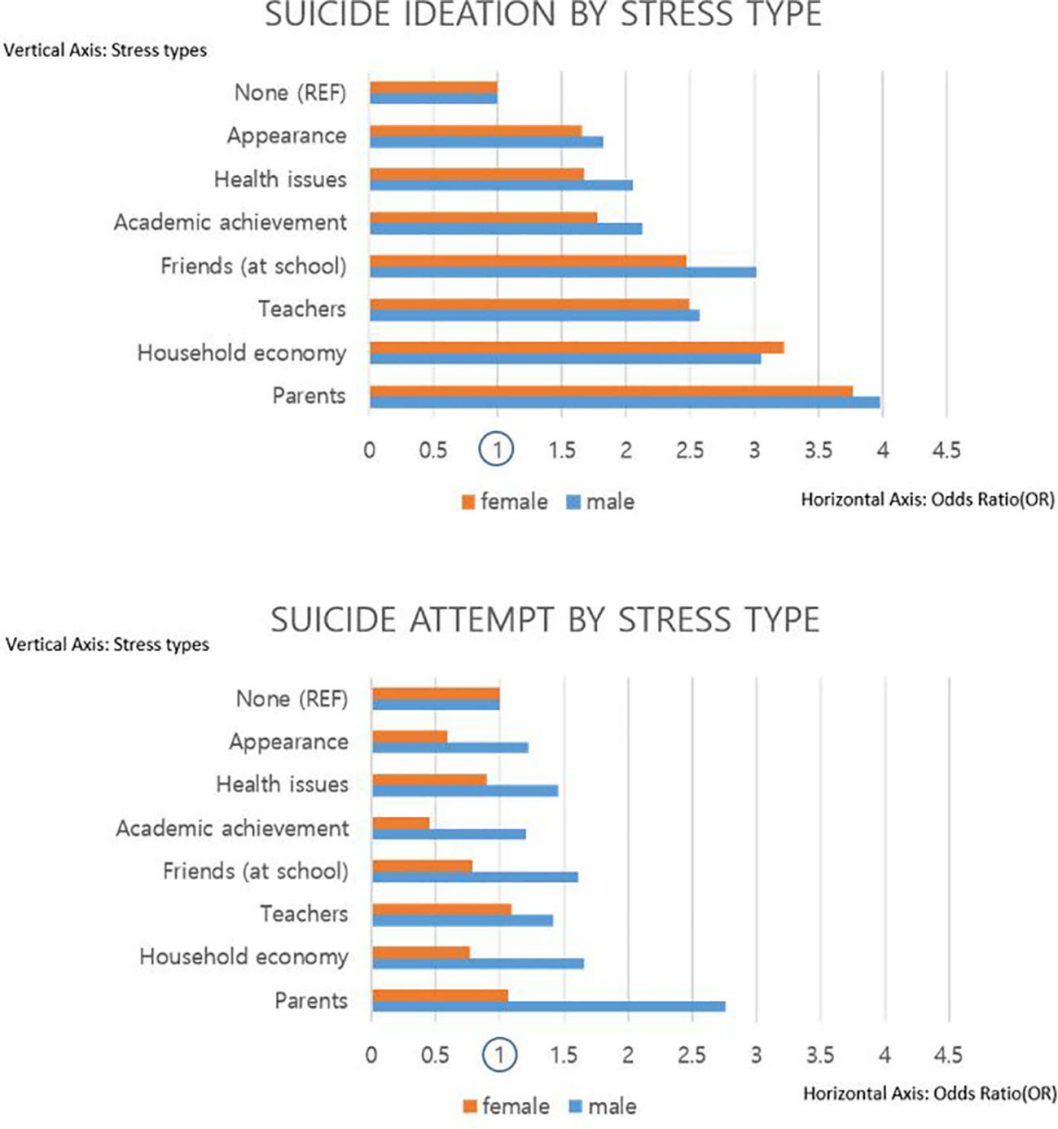
Suicide ideation and attempt by stress type stratified by sex.

The main independent variable of interest, in this study, was the type of stress adolescents experienced. From among the seven different reasons for stress—caused by parents, household economy, teachers, friends, academic achievement, health issues, and appearance—based on their commonality of sources, four groups were created—stress coming from home, school, academic achievement, and appearance(self). Later, each of these seven types of stress inducers were analyzed based on the variables of interest through subgroup analysis (**Figure 1**).

Covariates, such as sociodemographic and socioeconomic factors, health behaviors, and health conditions of this study’s participants were controlled. The sociodemographic factors were school grade levels (middle and high school), sex, and self-reported school grades (high, average, and low). The socioeconomic factors were parents’ education levels (under middle school, high school, and university or higher), living region (metropolitan and rural areas), and perceived household income (low, average, and high). Health behaviors included smoking or drinking habits (current and previous), and implementation of physical activities (none, 1 to 4 days a week, and 5 to 7 days a week). Mental health condition was adjusted with feelings of hopelessness (yes, no), given that the dependent variable was suicidal behavior. Lastly, the year variable (2015 and 2020) was adjusted.

### 2.4 Statistical analysis

To confirm the association between the types of stress inducers and suicidal behaviors—ideation and attempts—the covariates were compared by performing a chi-squared test. Multi logistic regression analysis was carried out for the main analysis. The results were reported using odds ratios (ORs) and confidence intervals (CIs). The data were analyzed and further stratified by sex, using SAS 9.4 (SAS Institute Inc; Cary, North Caroline). A P value <0.05 was considered to be statistically significant.

## 3 Results

**Table 1** shows the study population’s general characteristics. Of the 77,363 participants, 37,588 and 39,775 were male and female, respectively. The rate of participants who had seriously thought of committing suicide was noticeably higher in the female group than male group (13.2% versus 8.8%). From the groups with different types of stress, among those who had answered “yes” to suicidal ideation during the past 12 months, stress coming from family and home, or school and friends accounted for the highest rates in both the sexes (male: 15.2% and 13.7%, female: 25% and 20.4%). Nevertheless, in the academic achievement type of stress, the frequency was also the highest, regardless of sex (male: 21,639 out of 37,558, female: 25,094 out of 39,775). Among those who had tried committing suicide, the rate was 1.6% and 2.5% in male and female participants, respectively, and the same pattern (higher rate in female participants) was seen in suicidal ideation. The rate of participants who answered “yes” to suicidal attempts was markedly lower than ideation, but in the midst of the causes of stress, stress originating from family and home, or school and friends showed the highest rate. For ideation too, academic achievement had the most frequency as a stress inducer (male: 21,639 out of 37,588, female: 25,094 out of 39,775).

**Table 1:** General characteristics of the study population.

Variables	Suicidal Ideation											
	Male						Female					
	Total	Yes		No		*P-value*	Total	Yes		No		*P-value*
	N	N	%	N	%		N	N	%	N	%	
**Total (N=77,363)**	37,588	3,299	8.8	34,289	91.2		39,775	5,266	13.2	34,509	86.8	
**Stress Type**						<0.0001						<0.0001
None	2,147	49	2.3	2098	97.7		755	26	3.4	729	96.6	
Family and home	5,992	912	15.2	5,080	84.8		4,580	1,146	25.0	3,434	75.0	
School and friends	3,108	426	13.7	2,682	86.3		3,753	765	20.4	2,988	79.6	
Academic achievement	21,639	1,590	7.3	20,049	92.7		25,094	2,672	10.6	22,422	89.4	
Personal(appearance)	4,702	322	6.8	4,380	93.2		5,593	657	11.7	4,936	88.3	
**Year**						<0.0001						0.7102
2015	25,280	2,333	9.2	22,947	90.8		24,671	3,279	13.3	21,392	86.7	
2020	12,308	966	7.8	11,342	92.2		15,104	1,987	13.2	13,117	86.8	
**School year**						0.0179						0.4860
Middle school	18,098	1,523	8.4	16,575	91.6		19,091	2,536	13.3	16,555	86.7	
High school	19,490	1,776	9.1	17,714	90.9		20,684	2,730	13.2	17,954	86.8	
**Self-reported school grade**						<0.0001						<0.0001
High	16,072	1,251	7.8	14,821	92.2		16,559	1,958	11.8	14,601	88.2	
Average	10,317	848	8.2	9,469	91.8		11,926	1,371	11.5	10,555	88.5	
Low	11,199	1,200	10.7	9,999	89.3		11,290	1,937	17.2	9,353	82.8	
**Region**						0.0875						0.2777
Metropolitan city	19,418	1,695	8.7	17,723	91.3		20,848	2,748	13.2	18,100	86.8	
Town	16,517	1,482	9.0	15,035	91.0		16,839	2,263	13.4	14,576	86.6	
Rural	1,653	122	7.4	1,531	92.6		2,088	255	12.2	1,833	87.8	
**Father's education level**						0.0001						0.0057
Middle school or less	962	109	11.3	853	88.7		988	163	16.5	825	83.5	
High school	11,992	963	8.0	11,029	92.0		13,306	1,782	13.4	11,524	86.6	
College or above	24,634	2,227	9.0	22,407	91.0		25,481	3,321	13.0	22,160	87.0	
**Mother's education level**						0.1014						<0.0001
Middle school or less	768	83	10.8	685	89.2		824	152	18.4	672	81.6	
High school	14,328	1,232	8.6	13,096	91.4		16,282	2,159	13.3	14,123	86.7	
College or above	22,492	1,984	8.8	20,508	91.2		22,669	2,955	13.0	19,714	87.0	
**Household income**						<0.0001						<0.0001
Low	4,811	670	13.9	4,141	86.1		5,179	1,137	22.0	4,042	78.0	
Average	16,371	1,273	7.8	15,098	92.2		19,267	2,254	11.7	17,013	88.3	
High	16,406	1,356	8.3	15,050	91.7		15,329	1,875	12.2	13,454	87.8	
**Physical activity**						0.7089						<0.0001
None	9,935	862	8.7	9,073	91.3		17,845	2,228	12.5	15,617	87.5	
1-4 days a week	19,672	1,749	8.9	17,923	91.1		18,894	2,570	13.6	16,234	85.9	
5-7 days a week	7,981	688	8.6	7,293	91.4		3,036	468	15.4	2,568	84.6	
**Feeling of hopelessness**						<0.0001						<0.0001
Yes	7,368	2,275	30.9	5,093	69.1		11,387	3,921	34.4	7,466	65.6	
No	30,220	1,024	3.4	29,196	96.6		28,388	1,345	4.7	27,043	95.3	
**Alcohol Use**						<0.0001						<0.0001
Yes	16,247	1,791	11.0	14,456	89.0		12,878	2,482	19.3	10,396	80.7	
No	21,341	1,508	7.1	19,833	92.9		26,897	2,784	10.4	24,113	89.6	
**Cigarette Use**						<0.0001						<0.0001
Yes	8,520	1,108	13.0	7,412	87.0		2,994	799	26.7	2,195	73.3	
No	29,068	2,191	7.5	26,877	92.5		36,781	4,467	12.1	32,314	87.9	

Variables	Suicidal Attempt											
	Male						Female					
	Total	Yes		No		*P-value*	Total	Yes		No		*P-value*
	N	N	%	N	%		N	N	%	N	%	
**Total (N=77,363)**	37,588	593	1.6	36,995	98.4		39,775	1,003	2.5	38,772	97.5	
**Stress Type**						<0.0001						<0.0001
None	2,147	28	1.3	2,119	98.7		755	6	0.8	749	99.2	
Family and home	5,992	187	3.1	5,805	96.9		4,580	267	5.8	4,313	94.2	
School and friends	3,108	94	3.0	3,014	97.0		3,753	156	4.2	3,597	95.8	
Academic achievement	21,639	212	1.0	21,427	99.0		25,094	436	1.7	24,658	98.3	
Appearance	4,702	72	1.5	4,630	98.5		5,593	138	2.5	5,455	97.5	
**Year**						0.0002						0.0235
2015	25,280	441	1.7	24,839	98.3		24,671	657	2.7	24,014	97.3	
2020	12,308	152	1.2	12,156	98.8		15,104	346	2.3	14,758	97.7	
**School year**						0.1595						<0.0001
Middle school	18,098	303	1.7	17,795	98.3		19,091	548	2.9	18,543	97.1	
High school	19,490	290	1.5	19,200	98.5		20,684	455	2.2	20,229	97.8	
**Self-reported school grade**						<0.0001						<0.0001
High	16,072	208	1.3	15,864	98.7		16,559	334	2.0	16,225	98.0	
Average	10,317	145	1.4	10,172	98.6		11,926	263	2.2	11,663	97.8	
Low	11,199	240	2.1	10,959	97.9		11,290	406	3.6	10,884	96.4	
**Region**						0.4921						0.3226
Metropolitan city	19,418	292	1.5	19,126	98.5		20,848	547	2.6	20,301	97.4	
Town	16,517	274	1.7	16,243	98.3		16,839	410	2.4	16,429	97.6	
Rural	1,653	27	1.6	1,626	98.4		2,088	46	2.2	2,042	97.8	
**Father's education level**						0.0239						<0.0001
Middle school or less	962	24	2.5	938	97.5		988	49	5.0	939	95.0	
High school	11,992	170	1.4	11,822	98.6		13,306	358	2.7	12,948	97.3	
College or above	24,634	399	1.6	24,235	98.4		25,481	596	2.3	24,885	97.7	
**Mother's education level**						0.0291						<0.0001
Middle school or less	768	21	2.7	747	97.3		824	48	5.8	776	94.2	
High school	14,328	216	1.5	14,112	98.5		16,282	435	2.7	15,847	97.3	
College or above	22,492	356	1.6	22,136	98.4		22,669	520	2.3	22,149	97.7	
**Household income**						<0.0001						<0.0001
Low	4,811	121	2.5	4,690	97.5		5,179	251	4.8	4,928	95.2	
Average	16,371	205	1.3	16,166	98.7		19,267	382	2.0	18,885	98.0	
High	16,406	267	1.6	16,139	98.4		15,329	370	2.4	14,959	97.6	
**Physical activity**						0.0004						<0.0001
None	9,935	152	1.5	9,783	98.5		17,845	372	2.1	17,473	97.9	
1-4 days a week	19,672	277	1.4	19,395	98.6		18,894	507	2.7	18,387	97.3	
5-7 days a week	7,981	164	2.1	7,817	97.9		3,036	124	4.1	2,912	95.9	
**Feeling of hopelessness**						<0.0001						<0.0001
Yes	7,368	445	6.0	6,923	94.0		11,387	810	7.1	10,577	92.9	
No	30,220	148	0.5	30,072	99.5		28,388	193	0.7	28,195	99.3	
**Alcohol Use**						<0.0001						<0.0001
Yes	16,247	338	2.1	15,909	97.9		12,878	533	4.1	12,345	95.9	
No	21,341	255	1.2	21,086	98.8		26,897	470	1.7	26,427	98.3	
**Cigarette Use**						<0.0001						<0.0001
Yes	8,520	246	2.9	8,274	97.1		2,994	245	8.2	2,749	91.8	
No	29,068	347	1.2	28,721	98.8		36,781	758	2.1	36,023	97.9	

**Table 2** presents the results of logistic regression of factors associated with suicidal ideation and attempts, based on types of stress. With regard to suicidal ideation, every type of stress was highly associated with suicidal ideation and the family and home type of stress had the highest OR for both sexes (male: OR 3.81, 95% CI 2.81 to 5.15; female: OR 3.64, 95% CI 2.42 to 5.50). Moreover, the OR increased based on the order of appearance, academic achievement, school and friends, and family and home, as compared to the group that perceived no stress. Every type of stress was statistically significant and had a high OR in both male and female participants. Compared to the 2015 male participants, the 2020 ones were less likely to have suicidal ideation (OR 0.87, 95% CI 0.80 to 0.95). Conversely, only the female group that had to cope with stress from family and home, had suicidal attempts that were statistically significant with high ORs (OR 2.48, 95% CI 1.08 to 5.67). Attempted suicide was slightly different from suicidal ideation, in that, it showed that stress induced by academic achievement was less likely to be related to suicide attempts, representing the lowest OR in both sexes (male: OR 0.45, 95% CI 0.30 to 0.69; female: OR 1.21, 95% CI 0.53 to 2.75). Further, the male group’s ORs were statistically significant, while having reduced likelihood. A finding similar to suicide ideation was that compared to the 2015 participants, the 2020 ones were less likely to attempt suicide in the male group (OR 0.76, 95% CI 0.63 to 0.92).

**Table 2:** Results of factors associated with suicidal ideation and attempt by stress type.

Variables	Suicidal Ideation								Suicidal Attempt							
	Male				Female				Male				Female			
	OR	95% CI			OR	95% CI			OR	95% CI			OR	95% CI		
Stress Type																
None	1.00				1.00				1.00				1.00			
Family and home	3.81	2.81		5.15	3.64	2.42	-	5.50	1.01	0.67		1.54	2.48	1.08		5.67
School and friends	2.91	2.13	-	3.99	2.48	1.64	-	3.75	0.87	0.56	-	1.36	1.61	0.70	-	3.71
Academic achievement	2.13	1.58	-	2.87	1.77	1.18	-	2.66	0.45	0.30	-	0.69	1.21	0.53	-	2.75
Appearance	1.88	1.37	-	2.58	1.66	1.10	-	2.51	0.64	0.43	-	1.06	1.26	0.55	-	2.90
Year																
2015	1.00				1.00				1.00				1.00			
2020	0.87	0.80	-	0.95	0.97	0.91	-	1.04	0.76	0.63	-	0.92	0.87	0.76	-	1.00
School year																
Middle school	1.16	1.06	-	1.26	1.25	1.16	-	1.34	1.40	1.17	-	1.68	1.71	1.49	-	1.97
High school	1.00				1.00				1.00				1.00			
Self-reported school grade																
High	1.00				1.00				1.00				1.00			
Average	1.00	0.91	-	1.11	0.89	0.82	-	0.96	1.06	0.85	-	1.32	1.07	0.90	-	1.26
Low	1.09	0.99	-	1.20	1.06	0.98	-	1.15	1.27	1.04	-	1.56	1.17	1.00	-	1.38
Region																
Metropolitan city	1.00				1.00				1.00				1.00			
Town	0.99	0.91	-	1.07	0.99	0.93	-	1.06	1.05	0.89	-	1.25	0.87	0.76	-	1.00
Rural	0.84	0.68	-	1.03	0.92	0.79	-	1.07	1.08	0.72	-	1.63	0.82	0.60	-	1.12
Father's education level																
Middle school or less	0.96	0.75	-	1.24	0.81	0.65	-	1.00	1.02	0.63	-	1.66	1.14	0.80	-	1.64
High school	0.84	0.76	-	0.93	0.91	0.84	-	0.99	0.83	0.67	-	1.04	0.94	0.79	-	1.10
College or above	1.00				1.00				1.00				1.00			
Mother's education level																
Middle school or less	0.98	0.74	-	1.30	1.19	0.95	-	1.49	1.23	0.73	-	2.07	1.77	1.22	-	2.57
High school	0.98	0.89	-	1.08	0.95	0.88	-	1.03	0.94	0.77	-	1.16	1.08	0.92	-	1.27
College or above	1.00				1.00				1.00				1.00			
Household income																
Low	1.35	1.20	-	1.51	1.51	1.37	-	1.67	1.04	0.81	-	1.32	1.29	1.08	-	1.56
Average	0.95	0.87	-	1.04	0.95	0.88	-	1.03	0.78	0.64	-	0.95	0.80	0.69	-	0.93
High	1.00				1.00				1.00				1.00			
Physical activity																
None	1.21	1.07	-	1.35	0.94	0.84	-	1.07	0.91	0.73	-	1.15	0.65	0.52	-	0.80
1-4 days a week	1.12	1.01	-	1.24	0.96	0.85	-	1.08	0.78	0.64	-	0.95	0.75	0.61	-	0.92
5-7 days a week	1.00				1.00				1.00				1.00			
Feeling of hopelessness																
Yes	11.34	10.45	-	12.31	9.18	8.57	-	9.84	11.43	9.41	-	13.89	8.89	7.55	-	10.47
No	1.00				1.00				1.00				1.00			
Alcohol Use																
Yes	1.18	1.07	-	1.28	1.54	1.43	-	1.65	1.13	0.93	-	1.37	1.54	1.32	-	1.78
No	1.00				1.00				1.00				1.00			
Cigarette Use																
Yes	1.24	1.13	-	1.37	1.38	1.24	-	1.53	1.62	1.33	-	1.97	2.16	1.81	-	2.57
No	1.00				1.00				1.00				1.00			

**Table 3** presents numerical values of the subgroup analysis, stratified by the independent variables. For male participants, in all the covariates, the highest OR was observed when the family and home type of stress was the cause for suicidal ideation, with reference to adolescents with no stress inducers. For female participants, except fathers and mothers’ education levels being under middle school, which had the highest OR in stress coming from school and friends, every other covariate had the highest likelihood of suicidal ideation when stress emanated from family and home. The same tendency was observed in suicidal attempts. In fact, parents’ education levels affect suicidal behavior, as seen in **Table 3**. Interestingly, the lower the parents’ education levels, the less likely children are to have suicidal ideation and attempts. Compared to the group that perceived no stress, the overall ORs were above 1.00 (reference) in the groups where parents’ education levels were college or above. This trend is seen in male and female groups in both suicidal ideation and attempts.

**Table 3:** The results of subgroup analysis stratified by independent variables.

Variables	Suicidal Ideation																																	
	Stress type																																	
	Male																	Female																
	None	Family and home				School and friends				Academic achievement				Appearance				None	Family and home				School and friends				Academic achievement				Appearance			
	OR	OR	95% CI			OR	95% CI			OR	95% CI			OR	95% CI			OR	OR	95% CI			OR	95% CI			OR	95% CI			OR	95% CI		
School year																																		
Middle school	1.00	4.07	(2.65	-	6.25)	2.89	(1.84	-	4.53)	2.32	(1.52	-	3.54)	1.77	(1.13	-	2.78)	1.00	4.05	(2.33	-	7.04)	2.71	(1.55	-	4.74)	2.04	(1.18	-	3.52)	1.91	(1.10	-	3.34)
High school	1.00	3.43	(2.23	-	5.27)	2.82	(1.82	-	4.37)	1.90	(1.25	-	2.89)	1.94	(1.24	-	3.02)	1.00	3.11	(1.68	-	5.78)	2.16	(1.16	-	4.01)	1.47	(0.80	-	2.69)	1.37	(0.73	-	2.55)
Self-reported school grade																																		
High	1.00	4.20	(2.69	-	6.57)	3.07	(1.93	-	4.91)	2.24	(1.45	-	3.47)	1.75	(1.08	-	2.84)	1.00	5.14	(2.66	-	9.92)	3.22	(1.66	-	6.24)	2.47	(1.29	-	4.72)	2.11	(1.09	-	4.10)
Average	1.00	3.00	(1.72		5.23)	2.00	(1.12		3.57)	1.68	(0.98		2.89)	1.47	(0.82		2.64)	1.00	6.53	(2.36		18.08)	4.49	(1.61		12.47)	2.84	(1.04		7.82)	2.71	(0.98		7.55)
Low	1.00	4.36	(2.33	-	8.17)	3.79	(2.00	-	7.19)	2.52	(1.36	-	4.69)	2.48	(1.31	-	4.69)	1.00	1.56	(0.82	-	2.94)	1.14	(0.60	-	2.16)	0.83	(0.44	-	1.55)	0.82	(0.43	-	1.55)
Region																																		
Metropolitan city	1.00	3.78	(2.49		5.73)	2.84	(1.84		4.39)	2.16	(1.44		3.25)	1.86	(1.20		2.88)	1.00	4.09	(2.29		7.30)	2.62	(1.46		4.70)	1.99	(1.12		3.53)	1.97	(1.10		3.52)
Town	1.00	4.54	(2.77	-	7.44)	3.48	(2.09	-	5.77)	2.46	(1.51	-	4.00)	2.18	(1.31	-	3.65)	1.00	2.85	(1.58	-	5.14)	2.03	(1.12	-	3.66)	1.38	(0.77	-	2.48)	1.27	(0.70	-	2.29)
Rural	1.00	1.60	(0.56	-	4.60)	1.30	(0.44	-	3.79)	0.94	(0.34	-	2.57)	1.07	(0.35	-	3.26)	1.00	>0.001	<0.001	-	>999.999	>0.001	<0.001	-	>999.999	>0.001	<0.001	-	>999.999	>0.001	<0.001	-	>999.999
Father's education level																																		
Middle school or less	1.00	**0.71**	(0.23	-	2.19)	**0.64**	(0.20	-	2.09)	**0.50**	(0.17	-	1.52)	**0.46**	(0.13	-	1.61)	1.00	**0.79**	(0.19	-	3.39)	**0.99**	(0.23	-	4.35)	**0.66**	(0.16	-	2.75)	**0.64**	(0.15	-	2.79)
High school	1.00	2.66	(1.64		4.33)	2.19	(1.33		3.63)	1.44	(0.90		2.32)	1.44	(0.87		2.39)	1.00	3.12	(1.54		6.34)	2.08	(1.02		4.23)	1.46	(0.72		2.93)	1.32	(0.65		2.68)
College or above	1.00	**5.59**	(3.63	-	8.62)	**4.09**	(2.62	-	6.38)	**3.12**	(2.04	-	4.78)	**2.55**	(1.63	-	4.01)	1.00	**4.55**	(2.62	-	7.90)	**3.01**	(1.73	-	5.25)	**2.20**	(1.27	-	3.79)	**2.09**	(1.20	-	3.64)
Mother's education level																																		
Middle school or less	1.00	**0.70**	(0.23	-	2.18)	**0.56**	(0.16	-	1.98)	**0.38**	(0.12	-	1.18)	**0.41**	(0.11	-	1.51)	1.00	**0.91**	(0.15	-	5.66)	**1.04**	(0.17	-	6.52)	**0.56**	(0.09	-	3.39)	**0.44**	(0.07	-	2.82)
High school	1.00	3.08	(1.91	-	4.98)	2.72	(1.66	-	4.46)	1.86	(1.16		2.97)	1.90	(1.16		3.11)	1.00	4.27	(1.95	-	9.33)	2.97	(1.36	-	6.52)	2.12	(0.98		4.60)	1.86	(0.85		4.07)
College or above	1.00	**5.38**	(3.48	-	8.31)	**3.72**	(2.37	-	5.83)	**2.84**	(1.85	-	4.35)	**2.24**	(1.42	-	3.53)	1.00	**3.67**	(2.21	-	6.10)	**2.38**	(1.43	-	3.98)	**1.74**	(1.05	-	2.87)	**1.74**	(1.04	-	2.90)
Household income																																		
Low	1.00	2.85	(1.33	-	6.12)	2.47	(1.12	-	5.45)	1.55	(0.73	-	3.33)	1.71	(0.77	-	3.78)	1.00	2.54	(0.86	-	7.49)	2.03	(0.68	-	6.05)	1.51	(0.52	-	4.44)	1.40	(0.47	-	4.14)
Average	1.00	4.51	(2.58		7.87)	3.65	(2.06		6.45)	2.47	(1.43		4.27)	2.31	(1.30		4.09)	1.00	2.91	(1.54		5.49)	1.98	(1.05		3.75)	1.35	(0.72		2.52)	1.24	(0.65		2.34)
High	1.00	3.67	(2.42		5.55)	2.46	(1.59		3.80)	2.09	(1.40		3.13)	1.56	(1.00		2.43)	1.00	5.10	(2.72		9.58)	3.01	(1.60		5.67)	2.27	(1.22		4.21)	2.19	(1.16		4.12)

Variables	Suicidal Attempt																																	
	Stress type																																	
	Male																	Female																
	None	Family and home				School and friends				Academic achievement				Appearance				None	Family and home				School and friends				Academic achievement				Appearance			
	OR	OR	95% CI			OR	95% CI			OR	95% CI			OR	95% CI			OR	OR	95% CI			OR	95% CI			OR	95% CI			OR	95% CI		
School year																																		
Middle school	1.00	1.03	(0.57	-	1.85)	0.89	(0.47	-	1.68)	0.58	(0.33	-	1.04)	0.71	(0.37	-	1.34)	1.00	8.86	(1.23	-	64.00)	5.83	(0.80	-	42.37)	5.08	(0.71	-	36.51)	5.29	(0.73	-	38.37)
High school	1.00	1.04	(0.57	-	1.89)	0.85	(0.46	-	1.59)	0.36	(0.20	-	0.64)	0.65	(0.34	-	1.24)	1.00	1.14	(0.43	-	2.99)	0.74	(0.28	-	1.96)	0.47	(0.18	-	1.21)	0.47	(0.18	-	1.26)
Self-reported school grade																																		
High	1.00	0.71	(0.40	-	1.26)	0.54	(0.28	-	1.02)	0.32	(0.18	-	0.56)	0.39	(0.19	-	0.79)	1.00	1.99	(0.69	-	5.72)	0.99	(0.34	-	2.90)	0.97	(0.34	-	2.74)	0.84	(0.29	-	2.48)
Average	1.00	1.19	(0.49		2.89)	1.00	(0.39		2.55)	0.56	(0.23		1.34)	0.70	(0.27		1.85)	1.00	>0.001	<0.001	-	>999.999	>0.001	<0.001	-	>999.999	>0.001	<0.001	-	>999.999	>0.001	<0.001	-	>999.999
Low	1.00	1.85	(0.73	-	4.70)	1.74	(0.67	-	4.53)	0.76	(0.30	-	1.93)	1.40	(0.54	-	3.62)	1.00	1.75	(0.42	-	7.37)	1.14	(0.27	-	4.82)	0.86	(0.21	-	3.60)	0.84	(0.20	-	3.57)
Region																																		
Metropolitan city	1.00	1.00	(0.56		1.77)	0.94	(0.51		1.74)	0.44	(0.25		0.77)	0.58	(0.30		1.10)	1.00	2.43	(0.88		6.74)	1.21	(0.43		3.39)	1.07	(0.39		2.93)	1.22	(0.44		3.40)
Town	1.00	0.99	(0.52	-	1.86)	0.80	(0.41	-	1.56)	0.42	(0.23	-	0.79)	0.68	(0.34	-	1.34)	1.00	2.44	(0.58	-	10.22)	2.11	(0.50	-	8.88)	1.35	(0.33	-	5.60)	1.21	(0.29	-	5.12)
Rural	1.00	1.33	(0.15	-	12.07)	0.74	(0.07	-	7.35)	1.10	(0.13	-	9.14)	2.03	(0.22	-	18.67)	1.00	>0.001	<0.001	-	>999.999	>0.001	<0.001	-	>999.999	>0.001	<0.001	-	>999.999	>0.001	<0.001	-	>999.999
Father's education level																																		
Middle school or less	1.00	**0.80**	(0.36	-	1.76)	**0.85**	(0.38	-	1.92)	**0.45**	(0.21	-	0.97)	**0.76**	(0.34	-	1.72)	1.00	**0.61**	(0.09	-	4.27)	**0.46**	(0.06	-	3.42)	**0.33**	(0.05	-	2.30)	**0.18**	(0.02	-	1.46)
High school	1.00	1.80	(1.19		2.71)	1.80	(1.19		2.71)	0.54	(0.32		0.93)	0.73	(0.40		1.32)	1.00	4.74	(0.64		34.92)	2.90	(0.39		21.54)	2.30	(0.31		16.85)	2.26	(0.30		16.79)
College or above	1.00	**1.31**	(0.76	-	2.25)	**1.01**	(0.57	-	1.81)	**1.88**	(1.37	-	2.58)	**1.32**	(0.92	-	1.89)	1.00	**2.92**	(0.92	-	9.31)	**1.93**	(0.60	-	6.21)	**1.42**	(0.45	-	4.48)	**1.58**	(0.49	-	5.08)
Mother's education level																																		
Middle school or less	1.00	**0.18**	(0.05	-	0.74)	**0.33**	(0.07	-	1.45)	**0.04**	(0.01	-	0.24)	**>0.001**	<0.001	-	>999.999	1.00	**0.70**	(0.07	-	6.96)	**0.35**	(0.03	-	3.59)	**0.13**	(0.01	-	1.34)	**0.02**	(0.00	-	0.48)
High school	1.00	0.91	(0.44	-	1.90)	0.62	(0.28	-	1.37)	0.45	(0.22		0.93)	0.94	(0.44		2.00)	1.00	4.91	(0.67	-	35.84)	3.45	(0.47	-	25.30)	2.39	(0.33		17.36)	2.57	(0.35		18.88)
College or above	1.00	**1.40**	(0.79	-	2.50)	**1.26**	(0.69	-	2.32)	**0.59**	(0.33	-	1.04)	**0.66**	(0.34	-	1.26)	1.00	**2.73**	(0.85	-	8.74)	**1.72**	(0.53	-	5.58)	**1.46**	(0.46	-	4.63)	**1.56**	(0.48	-	5.05)
Household income																																		
Low	1.00	0.91	(0.31	-	2.68)	0.87	(0.28	-	2.72)	0.33	(0.11	-	1.00)	0.76	(0.24	-	2.42)	1.00	1.66	(0.21	-	13.00)	1.66	(0.21	-	13.18)	0.96	(0.12	-	7.51)	0.95	(0.12	-	7.56)
Average	1.00	1.17	(0.52		2.64)	1.09	(0.47		2.54)	0.59	(0.27		1.31)	0.82	(0.35		1.94)	1.00	2.20	(0.53		9.14)	1.30	(0.31		5.43)	1.04	(0.25		4.26)	1.14	(0.27		4.77)
High	1.00	0.97	(0.56		1.70)	0.71	(0.39		1.31)	0.41	(0.24		0.71)	0.53	(0.28		1.00)	1.00	3.30	(1.01		10.78)	1.73	(0.52		5.72)	1.42	(0.44		4.59)	1.46	(0.44		4.85)

**Table 4** is yet another independent subgroup analysis, focusing on the two years: 2015 and 2020, used in this study. Keeping 2015 as a reference, and by applying an interaction term between the years and the types of stress, the propensity of suicidal ideation and attempts were analyzed. In comparison to year 2015, both suicidal ideation and attempts decreased in year 2020. Nevertheless, only those whose stress came from parents or home had a higher tendency to attempt suicide, regardless of sex (male: OR 1.03, 95% CI 0.74 to 1.44; female: OR 1.06, 95% CI 0.81 to 1.4). **Figure 1** depicts the results of subgroup analysis among the independent variables, stratified by the variable of interest, which was divided into smallest units, on the basis of all seven answer choices to the question on: Which type of stress did participants suffer from the most? By comparing the two dependent variables, as well as visualizing it into a figure, the difference in results is clearer: suicide ideation is affected more by the type of stress, and suicide attempt was profoundly less likely to occur in the female group, compared to high ORs in ideation.

**Table 4:** Result of stress type in association with suicidal behavior by year.

Variables	Suicidal Ideation									Suicidal Attempt								
	2015	2020								2015	2020							
		Male				Female					Male				Female			
	OR	OR	95% CI			OR	95% CI			OR	OR	95% CI			OR	95% CI		
Stress Type																		
None	1.00	0.52	0.228	-	1.183	0.81	0.3	-	2.185	1.00	0.172	0.035	-	0.846	>0.001	<0.001		>999.999
Family and home	1.00	0.914	0.768	-	1.087	1.017	0.868	-	1.19	1.00	1.032	0.741	-	1.437	1.063	0.81	-	1.395
School and friends	1.00	0.852	0.643	-	1.131	0.828	0.679	-	1.009	1.00	0.673	0.379	-	1.198	0.755	0.509	-	1.119
Academic achievement	1.00	0.895	0.794	-	1.009	1.012	0.925	-	1.108	1.00	0.729	0.533	-	0.998	0.858	0.7	-	1.053
Appearance	1.00	0.724	0.551	-	0.951	0.883	0.735	-	1.062	1.00	0.707	0.411	-	1.217	0.727	0.504	-	1.047

## 4 Discussion

The present study used the group with no stress as a point of reference to investigate the types of stress experienced by adolescents and its association with suicidal behaviors, such as ideation and attempts. The findings revealed that suicidal ideation was significantly related to all types of stress—including family and home, school and friends, academic achievement—and manifested itself in both male and female participants. Whereas an increased likelihood of attempting suicide was seen only in the group whose stress emanated from family and home, as compared to the group that reported having no stress. Among the different types of stress, stress from family and home was also the highest in relation to suicidal attempts in the male group. Nevertheless, male participants who experienced stress mainly from academic achievements showed a decreased likelihood of committing suicide, and the results were statistically significant. A comparison of the two years, 2015 and 2020, revealed that in year 2020, the likelihood of suicidal ideation and attempts decreased for every stress cause, except stress from family and home.

Previous studies show a relationship between academic failure and suicidal behaviors in adolescents (24, 25). According to these studies, along with rapid industrialization and modernization come obstacles for social welfare, that are extremely challenging and demanding. They require the whole structural framework of education, including high school and the college entrance exams to be tempered, to ease the educational burden on students. In fact, this was not in alignment with this study, because **Table 2** shows that for suicidal attempts, the male group who picked academic achievement as the biggest cause for their stress, had decreasing OR, which was statistically significant.

This study also found that adolescents whose parents’ education levels were below middle school had decreased ORs compared to those whose parents had graduated from college or achieved education above that level, as shown in **Table 3**. This could relate to the context stated above relating to students’ academic pressure. Parents with high educational levels would expect their children to pursue similar or relatively higher levels of education, as compared with parents who were less educated (26, 27). These related studies indicate that parents’ education, occupation, and family economic status influence children’s studies, and are one of the most important factors, besides school. Moreover, it is likely that parents who have high education levels tend to be more strict and in control, and may have more conflict with their children while growing up (28, 29). Furthermore, this study also found that male adolescents were more likely to attempt suicide through **Figure 1**. According to WHO, completed suicide was 4 times more in male than in female in 2014 regarding the adolescents in Ireland. Also, while male are more likely to complete suicide, attempts are more frequently made in female (30). Previous studies claim that male are less sensitive in recognizing their stress or depressive mood caused by stress and neglect. It is called gender paradox, and this is also combined with male adolescents consciously concerned about the expected masculinity male should carry (31).

Relating the results with the theory that was mentioned in the introduction, as Human Birth Theory explains, the absence of affection from a caregiver, usually a parent, at the early stages of child’s life could cause the start of pathological cycle of mental disease that range from personality disorder to severe mood diseases, or even psychotic illnesses (9). Therefore, for those who have deficiency in non-conscious affection have hard time building a healthy relationship not only with parents, but also other human beings. This describes the result of this study; compared to those who do not get much stress, those who have stress coming from family and home have the highest odds of having suicidal ideas in both male and female, and second highest were those who get most stress from school and friends. In the basis of lack of vitality, these students have trouble forming a relationship with others, and the particular phase, adolescence, is a stage when these annulment trait outbursts into psychological problems, causing mood disorders or even psychotic illnesses.

As per this study’s results, stress from family and home were the most influential causes of stress that prompted adolescents to consider and commit suicide. According to a previous study, parent-child conflicts appear to be a salient precipitating factor for children who show suicidal behavior. Moreover, among children who committed suicide, family conflicts, particularly, parent-child conflicts were the most commonly reported precipitants. Many studies have discovered that conflicts within the family are the most crucial factors for children resorting to suicidal behavior (32–34). Going back to the human birth theory, those who had trouble receiving affection at the newborn period are likely to have trouble receiving it at the adolescence. After the outbreak of the coronavirus disease 2019, because of its highly contagious characteristics, many countries implemented regulations, such as social distancing or lockdowns. Many students went through substantial environmental changes in their daily lives, such as taking classes online and spending most of their time at home. Therefore, school closures may have affected adolescents’ mental health (35). Spending most time at home could be fatal for those in child abuse or severe neglect of children. Hence, there should be targeted policies to prevent adolescent suicides and find ways to manage those who have trouble with their families or home settings, especially in the pandemic era, when staying home is inevitable owing to social distancing restrictions.

There are several therapeutic interventions in order to treat the pathological conditions that may lead to self-harm in adolescence. One of the best known psychotherapy, psychodynamic psychotherapy has base theory that unconscious thoughts influence human behavior (36). Psychodynamic psychotherapies include therapeutic methods such as analysis of dreams, resistance, defense mechanisms (37). This method is focused on the cooperation between the therapist and the patient working together in order to understand the unconscious world of the patient. On the other hand, dialectical behavior therapy(DBT) includes not only individual psychotherapy, but also family as team consultation with therapist. Moreover, DBT has specific clinical focus on emotional dysregulation, self-harm, and interpersonal difficulties (38). These therapeutic methods were scientifically proven to be effective through numerous studies and experiments. The treatment and prevention methods for suicidal behaviors during adolescence continue to evolve, and there are many other approaches other than those stated above as well. It is crucial to understand the factors contributing to the development of suicidal behaviors in young individuals, thus personalized methods can be applied.

### 4.1 Limitations

This study had a few limitations. First, given the cross-sectional design, we were unable to track adolescents with different types of stress by individuals, over a continuous time period. Second, because the survey comprised self-reporting, it might have had untruthful answers, especially for suicidal attempts. Nevertheless, there are multiple previous studies using the same data, which successfully produced results that are reliable (39, 40). This very data were used in various research that investigated the association between one another. Thirdly, the secondary data was obtained from KYRBS and not collected by the researchers. Therefore, some variables that we wished to consider in the analysis were not included as they were unavailable in the data set. For example, when asked about the type of stress, in 2020, “other” was one of the options, and answers could be written in descriptive form. Therefore, in 2020, the number of participants decreased significantly because participants who chose the “other” had to be excluded. Lastly, this study did not investigate the psychopathology at the base of the suicidal ideation or behavior. Partially, depression was mentioned, but no other psychiatric disorders were discussed. This study was to search the association between stress types and suicidal behavior, so external stress for an adolescent was the main focus, but in the future, a detailed explanation of the mechanisms of how stress leads to suicidal behavior and related mental disorders should be provided.

### 4.2 Conclusion

Adolescents whose stress emanates from family and home, or school and friends, are more likely to consider or attempt suicide than those who have different causes of stress. Policies that target prevention of adolescent suicides are needed. Moreover, these policies must focus on finding ways to support those adolescents who have trouble in family or home settings, especially in the pandemic era when staying at home and social distancing is inevitable. Further studies should be conducted as the future cohort studies. Tracking down the same individual for the study period and comparing each groups would increase the reliability of the results.

## Data availability statement

Publicly available datasets were analyzed in this study. This data can be found here: https://www.kdca.go.kr/yhs/home.jsp Korean Adolescent Health Behavior Survey by Korean Center for Disease Control and Prevention. This study used data from the KYRBS, a secondary dataset accessible to the public, that does not include private material.

## Ethics statement

Ethical approval was not required for the study involving humans in accordance with the local legislation and institutional requirements. Written informed consent to participate in this study was not required from the participants or the participants’ legal guardians/next of kin in accordance with the national legislation and the institutional requirements.

## Author contributions

SK: Writing – original draft, Writing – review & editing. YP: Writing – review & editing. HJ: Writing – review & editing. E-CP: Writing – review & editing.
